# Deep Dyspareunia One Year After Nerve-Sparing Endometriosis Surgery: An Observational Study Highlighting Undesirable Outcomes

**DOI:** 10.3390/jpm16060307

**Published:** 2026-06-05

**Authors:** Nilton de Nadai Filho, Claudio Peixoto Crispi, Bruna Rafaela Santos de Oliveira, Claudio Peixoto Crispi, Marlon de Freitas Fonseca

**Affiliations:** 1 Department of Women’s Health—Fernandes Figueira National Institute for Women, Children and Youth Health—Oswaldo Cruz Foundation, Rio de Janeiro 22250-020, RJ, Brazil; nilton@niltonnadai.com.br (N.d.N.F.); brbrito14@gmail.com (B.R.S.d.O.); 2 Crispi Institute for Minimally Invasive Surgery; Rio de Janeiro 22640-102, RJ, Brazil

**Keywords:** endometriosis, dyspareunia, surgery, postoperative complications, patient outcome assessment, self report, follow-up studies, personalized medicine

## Abstract

**Background/Objectives:** This study evaluates the 1-year follow-up outcomes after minimally invasive nerve-sparing surgery for the complete excision of deep endometriosis (DE), with a specific focus on deep dyspareunia. Cases with undesirable outcomes were explored in detail to better understand the evolution of this cornerstone endometriosis-related symptom. This approach supports personalized medicine initiatives by seeking to stratify patients into likely surgical responders and non-responders. **Methods:** This is an interdisciplinary retrospective observational study assessing 195 consecutive cases. Inclusion criteria comprised women with an established diagnosis of DE who had been sexually active in the 6 months prior to surgery. Because pregnancy and postpartum can interfere with the longitudinal assessment of deep dyspareunia, women in these phases during follow-up were excluded. Additionally, individuals who had not been sexually active in the preceding 6 months for reasons unrelated to deep dyspareunia were excluded. Deep dyspareunia was measured using an 11-point (0–10) self-reported Numerical Rating Scale (NRS). Hierarchical clusters were established based on preoperative scores: NONE (NRS = 0), MILD (1 ≤ NRS ≤ 3), MODERATE (4 ≤ NRS ≤ 6), and SEVERE (NRS ≥ 7). **Results:** In the SEVERE cluster, 82.2% (95% CI: 72.4–92.0) of women improved by ≥3 points. In the NONE cluster, 70.1% (95% CI: 60.3–79.2) remained asymptomatic. Although improvements in deep dyspareunia were statistically significant across the total sample, individual trajectories were not uniform; the response was considered undesirable in 34 cases (17.4%; 95% CI: 12.1–22.8). The frequency of preoperatively asymptomatic women (NRS = 0) developing *De Novo* deep dyspareunia (NRS ≥ 3) at the 1-year follow-up was estimated at 14.9% (95% CI: 8.0–22.7). These results highlight the marked phenotypic and clinical heterogeneity in patient trajectories and the inherent unpredictability of adverse responses. **Conclusions:** Postoperative pain outcomes likely result from a complex interplay among surgical, myofascial, neurological, psychological, inflammatory, and hormonal factors. While surgery remains an effective and safe approach for treating pain, our findings underscore that even preoperatively asymptomatic patients should receive targeted counseling regarding the unexpected risk of developing postoperative deep dyspareunia.

## 1. Introduction

Endometriosis is a chronic disorder linked to endocrine, immunologic, proinflammatory, and proangiogenic processes. It affects approximately 10% of reproductive-age women and causes painful periods, chronic pelvic pain, deep dyspareunia, and infertility [1]. Currently, endometriosis is considered a debilitating condition that significantly affects women’s everyday lives, social relationships, sexuality, and mental health [2,3,4,5].

More than half of women with endometriosis report experiencing some degree of deep dyspareunia, which may lead to severe impairment of sexual function, relationships, and psychological well-being [6,7]. The treatment for deep dyspareunia may include the use of contraceptives, physical therapy, and lifestyle changes [8], but surgery has often been the chosen approach when medical treatment has not succeeded [9,10]. Endometriosis surgery frequently requires deep pelvic dissections for the complete excision of lesions. A qualified multidisciplinary team is necessary to minimize the risk of intestinal, urinary, and sexual complications after endometriosis surgery [11,12,13].

Endometriosis nerve-sparing surgery is known to improve endometriosis-related deep dyspareunia [14,15]. However, many studies evaluating surgical outcomes have excluded patients undergoing specific clinical treatments, patients with intestinal endometriosis, patients with infertility, and especially patients without deep dyspareunia prior to surgery [16,17,18]. In theory, these exclusions may represent a selection bias capable of limiting the generalization of the results. Furthermore, clinical before-and-after studies have usually been limited to simply comparing measures of central tendency to assess surgical efficacy, and this approach may obscure the risk of undesirable outcomes [19]. Indeed, many women become free of deep dyspareunia after endometriosis surgery. However, persistent deep dyspareunia may occur, preoperative deep dyspareunia may worsen, and the symptom may appear *De Novo* in women reporting no deep dyspareunia prior to surgery [20].

This study describes the 1-year follow-up of deep dyspareunia after minimally invasive nerve-sparing surgery for the complete excision of deep endometriosis (DE) lesions. The cases in which deep dyspareunia had undesirable outcomes over the first year were highlighted and individually explored in more detail to better understand its evolution after surgery. By characterizing the clinical course of deep dyspareunia over the first postoperative year, this study primarily seeks to identify potential predictors of poor surgical outcomes and formulate new hypotheses for future research.

## 2. Materials and Methods

### 2.1. Design, Setting, and Patients

Objectively, this is an interdisciplinary retrospective observational study utilizing a long-established database of clinical information collected for each of our endometriosis patients. The sample consists of consecutive patients referred by their personal gynecologists to the Crispi Institute for Minimally Invasive Surgery (ICRISPI)—a private institution located in Rio de Janeiro, RJ, Brazil. In this series, all surgeries were performed from January 2018 through September 2021 for consideration of minimally invasive surgical treatment of Deep Endometriosis (DE) for infertility and/or pain persisting after medical management; most were referred by their regular (continuity) gynecologists.

The inclusion criteria were women with an established diagnosis of DE (histopathological confirmation) who had been sexually active in the 6 months prior to surgery. Since pregnancy and the first postpartum year may interfere with the longitudinal assessment of deep dyspareunia, our exclusion criteria targeted women in these states at the time of follow-up. Additionally, we excluded individuals who had not been sexually active in the preceding six months for reasons unrelated to deep dyspareunia.

Although essentially descriptive, this study was conducted in two primary stages. Stage 1—Preliminary analytical phase: a before-and-after analysis comparing measures of central tendency was performed to confirm statistical improvements, and scatter plots were utilized to show a specific phenomenon: the occurrence of unfavorable (hidden) outcomes within the cohort. Stage 2—Exploratory semi-qualitative phase: cases exhibiting unfavorable outcomes were purposively selected and analyzed in detail to generate hypotheses regarding potential predictive factors. Given its exploratory nature, this study focused on the formulation of research hypotheses to guide future investigations.

Both the Strengthening the Reporting of Observational Studies in Epidemiology (STROBE) statement [21] and the updated Preferred Reporting of Case Series in Surgery (PROCESS) guidelines [22,23] were followed to enhance the quality of reporting.

### 2.2. Database and Preplanned Retrospective Data Collection

For more than ten years, ICRISPI has maintained standardized electronic databases to improve clinical documentation and support retrospective research. Under strict medical confidentiality, this comprehensive repository systematically tracks patient demographics, comorbidities, and diagnostic or laboratory findings from the initial preoperative visit. It also records detailed data on multidisciplinary team evaluations, prior pelvic surgeries (including available videos), longitudinal tracking of endometriosis-related pain, and detailed descriptions of performed procedures.

Preoperative, surgical, and postoperative follow-up data for this cohort were collected through retrospective chart abstraction and a standardized database (detailed below). Two experienced, postgraduate-trained research nurses performed the data abstraction, with all entries double-checked to ensure accuracy.

### 2.3. Surgical Protocol

Following outpatient preoperative evaluations, the surgical indication was confirmed by the attending gynecologist (C.P.C.), who subsequently led all procedures—a senior physician with over 20 years of expertise in DE surgery. With very few exceptions, the specialized surgical team (gynecologist, proctologist, urologist, nurse, and anesthesiologist) was identical in each instance.

In summary, endometriotic lesions were completely excised via laparoscopy using the nerve-sparing technique described in our previous study on 6-month deep dyspareunia outcomes [20]. This surgical strategy remained consistent over the ten-year period. All procedures (whether robot-assisted or conventional laparoscopy) began with a thorough abdominal cavity inspection to assess and remove lesions identified preoperatively by physical examination and magnetic resonance imaging. At the conclusion of each surgery, an intraoperative cystoscopy was routinely performed to verify suture integrity, confirm ureteral patency, and rule out thermal bladder injury. Hysteroscopy was occasionally utilized for uterine pathology or infertility investigations. Excised specimens were individually labeled for histopathological analysis, which provided the definitive diagnosis of DE.

Surgical data were collected using specific standardized instruments that automatically entered information into the patient’s medical record. These instruments captured general data (first puncture technique, type of anesthesia, duration, and estimated blood loss) and specific findings from the cavity inventory, classified by topography (upper abdomen, as well as the anterior, posterior, and lateral compartments of the pelvis). The description of the surgery is also standardized, detailing resected structures, along with any concurrent non-endometriosis procedures (e.g., cholecystectomy, myomectomy, hysteroscopy). When necessary, surgical videos were reviewed by a staff surgeon to ensure maximum accuracy.

Postoperative complications were graded according to the Clavien-Dindo classification, with grades I–II defined as minor and grades III–V as major [24].

### 2.4. Deep Dyspareunia Assessment

During the preoperative consultation, all patients were thoroughly evaluated for both menstrual and nonmenstrual endometriosis-related pain symptoms, including deep dyspareunia, dysmenorrhea, and pelvic pain. Each symptom was measured on an 11-point (0–10) self-reported numerical rating scale (NRS), with patients instructed to report the intensity of pain experienced over the last 6 months. According to the ICRISPI’s regular follow-up program, patients were invited for two post-operative clinical evaluations using the same methodology as the preoperative assessment: at 6 months and 1 year.

To systematically evaluate deep dyspareunia (the primary outcome of this study), we initiated the clinical interview with a two-part question: “Have you had pain during sexual intercourse in the last six months? If yes, is this pain at the beginning of penetration or during deep penetration?” Focusing strictly on deep vaginal penetration, participants recorded their pain scores on the NRS, while those without sexual activity in the previous six months checked ‘not applicable.’ The protocol explicitly encouraged the documentation of any form of vaginal penetration—whether same-gender, opposite-sex, or involving sex toys. Superficial dyspareunia fell outside the scope of this evaluation.

**Figure 1:**
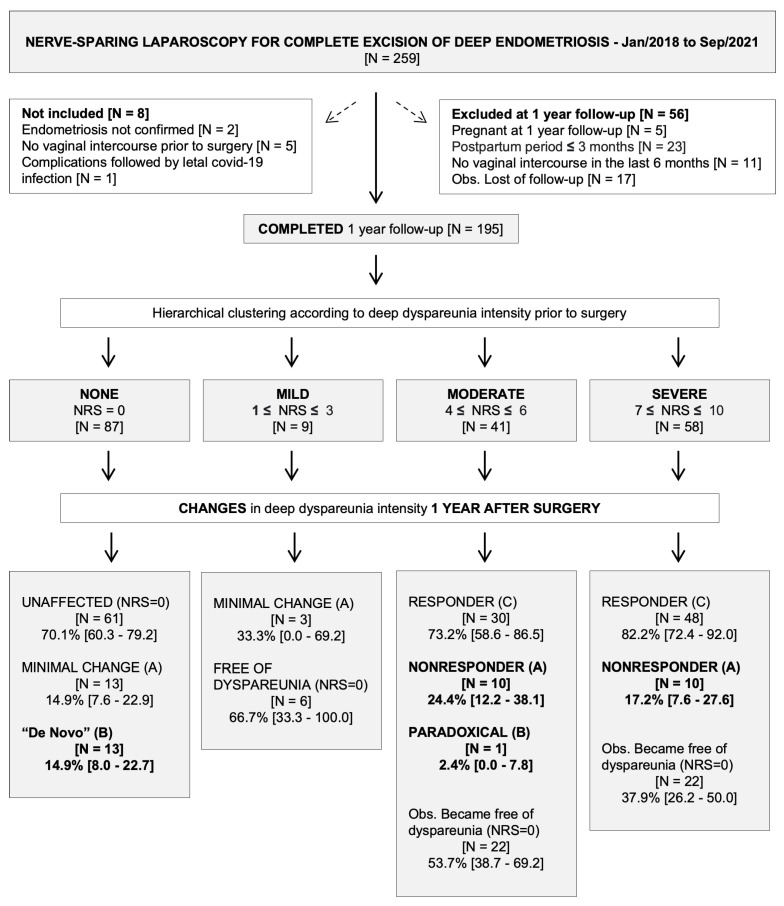
Response of deep dyspareunia intensity (primary outcome) to minimally invasive nerve-sparing complete excision of endometriosis at 1-year follow-up. Deep dyspareunia was assessed on a self-reported 11-point Numeric Rating Scale (NRS). (A): NRS changed by less than 3 points. (B): NRS ↑ by 3 points or more. (C): NRS ↓ by 3 points or more. Frequency expressed as percentage [95% confidence interval]. The primary exclusion criterion was pregnancy or the postpartum period, followed by a lack of sexual intercourse during the second postoperative semester due to personal reasons unrelated to pain.

Patient expectations regarding surgical outcomes naturally vary based on their baseline symptoms; asymptomatic individuals aim to maintain their status, whereas those experiencing significant pain anticipate clinical improvement. Consequently, this study established hierarchical clusters based on preoperative deep dyspareunia scores: NONE (NRS = 0), MILD (1 ≤ NRS ≤ 3), MODERATE (4 ≤ NRS ≤ 6), and SEVERE (NRS ≥ 7) (Figure 1). Individual NRS scores are reported as integers, reflecting the original scale; decimal places are maintained exclusively for median values.

In summary, the postoperative response was initially assessed by comparing scores in a non-experimental observational before-and-after study (Null Hypothesis: The median of differences between scores prior to and at follow-up equals 0). Then, cases where deep dyspareunia was not satisfactorily improved (or even worsened) over the first year were highlighted and explored in more detail through an individualized semi-qualitative approach. For this, as in previous studies, a minimum difference of 3 points on the NRS was considered the threshold for a clinically relevant response to endometriosis treatment [20,25].

### 2.5. Statistics

Charts and statistics were developed using IBM^®^ SPSS^®^ Statistics Version 29.0.0.0-241 (IBM Corp., Armonk, NY, USA). The nonparametric independent-samples Mann-Whitney U test was used to compare groups according to ordinal variables. Pearson’s Chi-square test (or Fisher’s Exact Test) was used to compare groups according to categorical variables. The non-parametric related-samples Wilcoxon signed-rank test was used for before-after comparison. The statistical results were considered significant when *p* < 0.05 (2-sided).

## 3. Results

### 3.1. Cohort Profile

A total of 259 women were screened for the study, of whom 195 (75.29%; CI 95%: 69.71–80.12) met the eligibility criteria and completed the 1-year postoperative clinical follow-up. Of the 195 subjects included, 108 (55.4%; CI 95%: 48.2–62.6) had reported some form of deep dyspareunia prior to surgery (NRS > 0). The main reasons for exclusion included pregnancy or the postpartum period at the 1-year follow-up (N = 28; 14.4%; CI 95%: 9.2–19.5). Among all evaluated women, 11 had not engaged in sexual intercourse during the second postoperative semester due to personal reasons unrelated to pain. Of the 259 women initially selected, 17 were lost to follow-up due to non-response, representing a rate of 6.56% (95% CI: 3.56–9.56%). The patient screening and selection flowchart, detailing inclusion and exclusion criteria, is presented in Figure 1.

Overall, this cohort comprised healthy Brazilian women who were occasional drinkers, non-smokers, not obese, with a higher education level, and middle-class income. Given the exploratory nature of this study and the small size of the MILD cluster (N = 9), the sample was dichotomized based on preoperative deep dyspareunia severity. This approach enabled a preliminary comparison between women with minimal (NONE/MILD) and those with significant pain (MODERATE/SEVERE). When the cohort was dichotomized, women with NONE or MILD deep dyspareunia (NRS ≤ 3) were statistically older (*p* = 0.002; Mann-Whitney U test) than those with MODERATE or SEVERE deep dyspareunia (NRS > 3). Additionally, the groups were statistically different in terms of ethnicity (*p* = 0.03; Pearson Chi-square test): the NONE or MILD deep dyspareunia group had a higher proportion of individuals of European descent and a lower proportion of individuals of African descent compared to the MODERATE or SEVERE group. Demographic characteristics of the sample are presented in Table 1. Individual raw data can be accessed in the Supplementary File.

Concerning postoperative complications, there were no cases of anastomotic leakage nor any grade III or IV Clavien-Dindo complication in this series.

### 3.2. Deep Dyspareunia over the First Year After Surgery

Approximately 6 months after surgery, 170 women underwent an intermediate exploratory evaluation: 100 women (58.8%) reported NONE, 33 (19.4%) reported MILD, 19 (11.2%) reported MODERATE, and 18 (10.6%) reported SEVERE deep dyspareunia. Subsequently, the 1-year follow-up assessment was conducted as the primary outcome of this study. For the convenience of the participants, the 1-year follow-up did not occur precisely 12 months after surgery in all cases; the median was 12.8 months (25th–75th percentile: 12.2–14.3).

**Figure 2:**
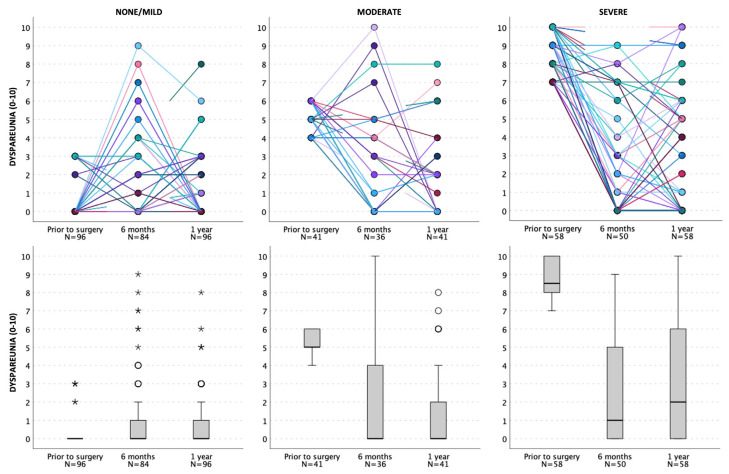
Individual changes in deep dyspareunia after minimally invasive nerve-sparing complete excision of endometriosis over the first year of follow-up. Deep dyspareunia was assessed using a self-reported 11-point Numeric Rating Scale (NRS) on three occasions: prior to surgery (during the preoperative evaluation period), at 6 months (intermediate evaluation), and at 1-year follow-up (main outcome). In this illustration, the self-reported deep dyspareunia intensity prior to surgery was used to group the analysis into three hierarchical clusters: NONE (NRS = 0)/MILD (1 ≤ NRS ≤ 3), MODERATE (4 ≤ NRS ≤ 6), and SEVERE (NRS ≥ 7). The missing data at 6 months are displayed as blank gaps in rows. Circles (outliers) represent values that lie outside of the following ranges: 3rd quartile + 1.5 x interquartile range OR 1st quartile—1.5 x interquartile range. Stars (extreme outliers) represent values that lie outside of the following ranges: 3rd quartile + 3 x interquartile range OR 1st quartile—3 x interquartile range.

The cohort as a whole (N = 195) demonstrated a statistically significant improvement in deep dyspareunia scores 1 year after surgery: the median NRS (25th–75th percentile) decreased from 4 (0–9) to 0 (0–6) (*p* < 0.001; Wilcoxon signed rank test). However, analyzing the clusters separately enabled a more detailed exploration of this phenomenon. For example, when each cluster was evaluated using a scatter plot, it became evident that the individual trends observed in deep dyspareunia scores were not uniform over the first year after surgery. Indeed, in some cases, there were significant transient changes at 6 months. The responses regarding deep dyspareunia observed over the first year after surgery are summarized in Figure 2.

### 3.3. Classifying the Responses to Surgery at 1-Year Follow-Up

Patients in the NONE cluster who reported no deep dyspareunia at the 1-year follow-up were labeled “Unaffected,” whereas those who reported an increase of at least 3 points in their deep dyspareunia score (a clinically relevant change) were labeled as presenting *De Novo* deep dyspareunia, an undesirable outcome. Patients in the NONE or MILD cluster who showed less than a 3-point increase in their deep dyspareunia scores were labeled as having “Minimal Change.”

Patients in the MODERATE or SEVERE clusters who reported an improvement in deep dyspareunia severity of 3.0 or more points were labeled “Responders,” whereas those with improvements of less than 3.0 points were labeled “Nonresponders.” One patient in the MODERATE cluster experienced relevant worsening of deep dyspareunia at the 1-year follow-up (preoperative NRS = 5; NRS at 6 months = 8; NRS at 1 year = 8); her response was labeled “Paradoxical”, an undesirable outcome.

### 3.4. Women with None or Mild Deep Dyspareunia Prior to Surgery

The NONE (N = 87) and MILD (N = 9) clusters showed a weak but statistically significant change in NRS scores when assessed together (N = 96) at the 1-year follow-up compared with scores prior to surgery (*p* = 0.004; Wilcoxon signed rank test). In fact, although 70 of these patients (72.9%) reported some improvement or maintained their exact same low scores (7 and 63 patients, respectively), 26 patients (27.1%) reported some undesirable response (Figure 2).

**Figure 3:**
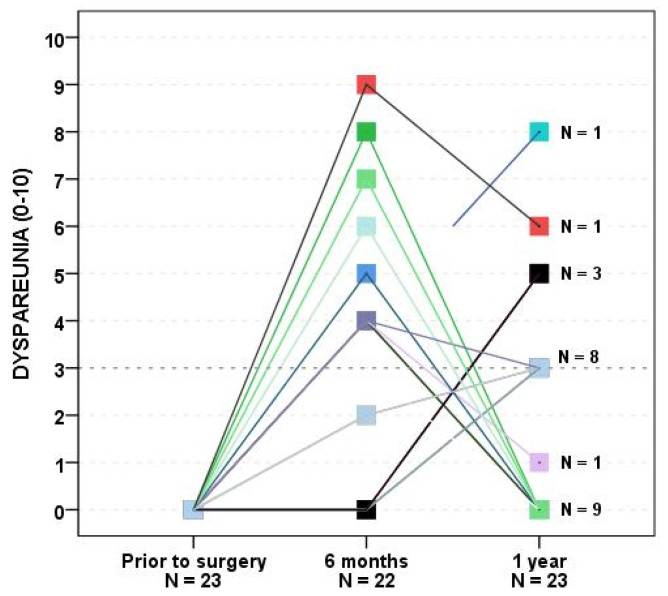
Individual evaluation of deep dyspareunia development following minimally invasive nerve-sparing complete excision of endometriosis. The chart displays the Numerical Rating Scale (NRS) scores for deep dyspareunia in the 23 women who reported no deep dyspareunia prior to surgery but developed it (even transiently) afterward (*De Novo*). Deep dyspareunia was assessed using an 11-point self-reported NRS at three time points: preoperatively, at 6 months (intermediate evaluation), and at 1 year (main outcome). Only patients whose NRS score increased by 3 or more points at either the 6-month or 1-year follow-up are included in this chart. Patient Case 2 shows a discontinuity in the line graph due to the lack of an available 6-month evaluation.

Separate assessment of the 87 patients in the NONE cluster revealed that 12 women had reported relevant deep dyspareunia (NRS ≥ 3) at the 6-month follow-up. However, only one of these 12 patients (case number 1) continued to report relevant deep dyspareunia at the 1-year follow-up (NRS at 6 months = 9; NRS at 1 year = 6). Notably, a different group of 13 patients from the NONE cluster reported some relevant deep dyspareunia at the 1-year follow-up (Figure 3).

The frequency of patients with no baseline deep dyspareunia (NRS = 0; NONE Cluster) remaining unaffected 1 year after surgery was estimated at 70.1% (95% CI: 60.3–79.2). Meanwhile, the frequency of *De Novo* deep dyspareunia at the 1-year follow-up within this same cluster was estimated at 14.9% (95%CI: 8.0–22.7).

### 3.5. Women with Moderate Deep Dyspareunia Prior to Surgery

In the cluster MODERATE (N = 41; median NRS prior to surgery = 5.0), there was a significant improvement in deep dyspareunia scores after 1 year compared with scores prior to surgery (*p* < 0.001; Wilcoxon signed rank test). A clinically relevant improvement in deep dyspareunia (≥3 points) was reported by 30 patients (Responders), a worsening (Paradoxical) was reported by 1 patient (preoperative NRS: 5; NRS at 6 months = 8; NRS at 1 year = 8), and no clinically relevant improvement was reported by 10 women (Nonresponders).

A total of 36 subjects (87.8%) of the MODERATE cluster attended the 6-month follow-up consultation. The median NRS (25th–75th percentile) of deep dyspareunia for the MODERATE cluster at 6 months was 0.0 (0.0–4.0). There were only 3 cases of clinically worsening at the 6-month follow-up in the MODERATE cluster, with only one case of worsening persisting at the 1-year follow-up.

Among women with moderate deep dyspareunia (4 ≤ NRS ≤ 6), the frequency of clinical benefit from this surgery—defined as an improvement of ≥ 3 points 1 year postoperatively—was estimated at 73.2% (95% CI: 58.6–86.5).

### 3.6. Women with Severe Deep Dyspareunia Prior to Surgery

In the SEVERE deep dyspareunia cluster (N = 58; median NRS score prior to surgery = 8.5), there was a statistically significant improvement in scores after 1 year (*p* < 0.001; Wilcoxon signed rank test). Clinically relevant improvements in deep dyspareunia (≥3 points) were reported by 48 women (Responders), whereas 5 women reported no change *in their scores and 1 woman reported worsening deep dyspareunia (preoperative NRS: 9; NRS at 6 months = 8; NRS at 1 year = 10).

A total of 50 women (86.2%) of the SEVERE cluster were assessed at the 6-month visit. The median NRS (25th–75th percentile) of deep dyspareunia of the SEVERE cluster at 6 months was 1 (0–5). There were two cases of worsening in the severe cluster at 6 months follow-up, but none were clinically relevant changes (worsening < 3 points). At the 6-month follow-up, we found 10 cases of insufficient response (deep dyspareunia reduction < 3 points). We still observed 20 cases that had completely recovered from deep dyspareunia at the 6-month visit (NRS = 0), with 12 remaining free of deep dyspareunia at the end of the first year of follow-up. The frequency of women with severe deep dyspareunia (NRS > 7) benefiting from this type of surgery with an improvement of ≥3 points at the 1-year follow-up was estimated at 82.8% (95%CI: 72.4–92.0).

### 3.7. When the Result of Surgery Is Frustrating at 1-Year Follow-Up

Regarding deep dyspareunia at 1-year follow-up, the responses were considered undesirable in 34 cases (17.4%; 95%CI: 12.1–22.8). Thus, due to the limited number of individuals, these cases were analyzed in detail through a semi-qualitative individualized approach instead of statistical tests. In these 34 selected cases, the main reason for surgical intervention was pelvic pain (21 patients; 61.8%). Pain associated with infertility was the indication of surgery for 7 patients (20.6%), and infertility alone was the most important complaint in 2 patients (5.9%). The main anatomical sites affected by endometriosis were the uterosacral ligaments (30 cases; 88.2%), ovarian fossa (27 cases; 79.4%), and parametrium (23 cases; 67.7%). Nine of these 34 patients had adenomyosis (26.5%); 2 of them underwent total hysterectomy. Details of their surgical procedures (including the main sites of endometriosis lesions) are shown in Table 2.

In the individual assessment, noteworthy clinical conditions were identified in 5 of the 34 selected patients: endometritis (N = 1), history of pelvic inflammatory disease (N = 1), vaginismus (N = 1), vulvodynia (N = 1), and interstitial cystitis (N = 1). Regarding hormone use after surgery, 10 patients received Goserelin at some point during the first year, whereas 16 subjects were using no hormone therapy at the 12-month visit. Still considering these 34 selected cases, 4 patients had a previous diagnosis of major depression, which might also act as a confounding factor. The evolution of their main painful symptoms and other potential confounding factors is detailed in Table 3.

**Figure 4:**
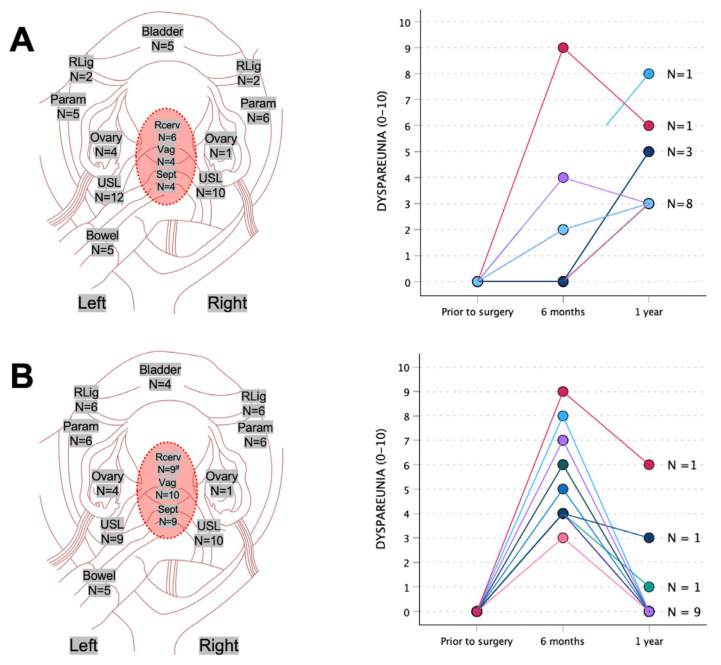
Distribution of the main endometriosis lesions in patients who developed *De Novo* deep dyspareunia over the first year of follow-up after minimally invasive nerve-sparing complete excision of endometriosis. (**A**) Patients who developed *De Novo* deep dyspareunia at 1 year of follow-up (N = 13). (**B**) Patients who developed *De Novo* deep dyspareunia at 6 months of follow-up (N = 12). RLig: Round Ligament; Param: Parametrium; USL: Uterosacral Ligament; Rcerv: Retrocervical area; Vag: Vagina; Sept: Rectovaginal Septum; Bowel: rectum and/or sigmoid. One patient (case number 2) was displayed as a discontinuity in the line because she was not evaluated at 6 months.

The distribution of the endometriosis lesions in the pelvis of the patients who developed *De Novo* deep dyspareunia over the first year of follow-up is presented in Figure 4. Considering the retrocervical region, vagina, and rectovaginal septum (sites of direct contact during sexual intercourse), endometriosis lesions were less frequent in the subgroup of women with *De Novo* deep dyspareunia at the 1-year follow-up than in those with transient *De Novo* deep dyspareunia at the 6-month follow-up.

Among the 9 Nonresponders in the MODERATE cluster (N = 41), endometriosis lesions were distributed as follows: retrocervical lesions in 88.9% (N = 8/9; 95%CI: 56.5–98.0), vaginal involvement in 55.6% (N = 5/9; 95%CI: 26.7–81.1), and intestinal, pelvic floor muscle, and rectovaginal septum involvement in 22.2% each (N = 2/9; 95%CI: 6.3–54.7). Conversely, for the 11 Nonresponders in the SEVERE cluster (N = 58), the prevalence of lesions included: retrocervical involvement in 81.8% (N = 9/11; 95%CI: 48.2–97.7), vaginal and intestinal involvement in 36.4% each (N = 4/11; 95%CI: 14.9–65.9), and rectovaginal septum involvement in 9.1% (N = 1/11; 95%CI: 0.5–37.9).

## 4. Discussion

### 4.1. Overview

This study evaluated the deep dyspareunia outcomes of 195 consecutive cases of sexually active women who received minimally invasive nerve-sparing complete excision of endometriosis. Overall, although there were significant improvements in deep dyspareunia at 1-year follow-up, the individual observations were not uniform and the response was considered undesirable in 34 cases (17.4%; 95%CI: 12.1–22.8), including Nonresponders (improvements < 3 points; N = 20), Paradoxical response (worsening of pain; N = 1) and *De Novo* deep dyspareunia (emergence of DDyspareuna that did not exist prior to surgery; N = 13). Individual analysis of these 34 cases using a semi-qualitative approach failed to identify predictors of undesirable outcomes regarding deep dyspareunia. Therefore, we have hypothesized that postoperative pain outcomes result from a complex interplay among surgical, myofascial, neurological, psychological, inflammatory, and hormonal elements.

### 4.2. De Novo Deep Dyspareunia at 6 Months Follow-Up

Regarding the 87 patients without deep dyspareunia prior to surgery (NRS = 0), 12 reported *De Novo* deep dyspareunia at the 6-month follow-up; however, only two of them continued to report relevant deep dyspareunia at the 1-year mark. Thus, an individual qualitative analysis revealed that a subgroup of patients experienced transient *De Novo* deep dyspareunia at 6 months (N = 12), and these women were largely different from those reporting *De Novo* deep dyspareunia at the 1-year follow-up (N = 13). These findings corroborate the idea that long-term follow-up should be considered for evaluation of deep dyspareunia in asymptomatic women because its evolution over time has not been predictable or, much less, linear.

Elevated inflammatory markers, such as interleukin-6 and C-reactive protein, along with insulin resistance, are well-established components of the metabolic response to surgical trauma [26]. Furthermore, although laparoscopic hysterectomy exerts a lower neuroendocrine and inflammatory impact than laparotomic surgery [27], it is critical to note that endometriosis foci exhibit a greater density of nerve endings, a lower threshold for nerve excitability, and altered pain conduction pathways [28]. Therefore, we hypothesize that transient *De Novo* deep dyspareunia at the 6-month follow-up may correlate with natural local tissue repair mechanisms and the metabolic response to surgical trauma. Indeed, the hypothesis of a correlation between the local inflammatory reaction and *De Novo* deep dyspareunia at 6 months is consistent with the concentration of endometriosis lesions in the rectovaginal septum, retrocervical, and uterosacral ligaments in this group of patients (Figure 4). This parallel is further supported by literature demonstrating a statistical association between the excision of posterolateral parametrial endometriosis and postoperative deep dyspareunia or sexual dysfunction [29].

### 4.3. De Novo Deep Dyspareunia at 1 Year Follow-Up

One of the primary considerations when a patient reports worsening pelvic pain after surgery is treatment failure resulting from incomplete surgical excision, leading to the recurrence of endometriosis. In this series, however, deep dyspareunia did not intensify in parallel with other symptoms during the course of the first year—such as dysmenorrhea or non-menstrual pelvic pain—as would be expected if endometriosis recurrence were the primary driver (Table 3). Among women who did not experience deep dyspareunia before surgery, 14.9% (95% CI: 8.0–22.7%) reported *De Novo* deep dyspareunia at the 1-year follow-up. Overall, the presentation of *De Novo* deep dyspareunia throughout the first post-operative year occurred concurrently with an improvement in other painful symptoms, such as dysmenorrhea or non-menstrual pelvic pain. Most patients who developed *De Novo* deep dyspareunia within 1 year were not affected by endometriosis in the rectovaginal septum and/or vagina (Figure 4, Table 2). This finding is consistent with an earlier study restricted to women with severe deep dyspareunia [30], which noted that patients without anatomical endometriosis in the rectovaginal septum also experienced a recurrence of deep dyspareunia during the first year after surgery.

While the anatomical location of surgically resected endometriosis lesions is a major determinant of postoperative pain patterns, it may not be the sole factor explaining the recurrence or onset of symptoms. Neurogenic inflammation and viscero-visceral convergence, both well-documented in endometriosis, may account for the clinical presentation of inflammatory and visceral pain even in the absence of anatomical endometriotic foci within the bladder or intestines [31]. This pattern of somato-visceral convergence may interact dynamically with pelvic floor myofascial hypertonicity, a condition closely associated with deep dyspareunia [28]. Concerning mental disorders and psychiatric conditions, depression and pain have been associated with central pain sensitization, sharing biological pathways and neurotransmitters [32]. In fact, the available evidence indicates that psychological interventions are effective in improving the pain, quality of life, and mental health variables of women with endometriosis [33]. In this series, the existence of major depression was verified in 4 patients with undesirable response at 1 year of follow-up (Table 3). As suggested in previous studies [20,34], mental disorders should also be considered as potential cofactors in the development of deep dyspareunia as well as in other pain conditions.

### 4.4. Limitations and Strengths

While randomized clinical trials represent the gold standard for evaluating treatment efficacy, they face significant practical and ethical challenges in complex surgical scenarios such as endometriosis excision. Therefore, although the retrospective, observational design of this study could be viewed as a limitation, our interdisciplinary framework operates much like an early-stage safety trial. It prioritizes not only symptom improvement but also critically evaluates previously overlooked risks—such as *De Novo* deep dyspareunia. By tracking patients without prior deep dyspareunia through a longitudinal cohort, we emphasize that surgical outcomes must be assessed comprehensively, carefully balancing expected therapeutic benefits against potential postoperative harms. Yet, when assessing surgical complications, much attention is usually paid to fistulas, dehiscence, urinary or intestinal dysfunction [35,36,37], and follow-up studies frequently do not highlight undesirable outcomes in patients who had been asymptomatic before surgery. This approach supports personalized medicine initiatives by seeking to stratify patients into likely surgical responders and non-responders.

Follow-up analysis included the majority of eligible participants. Of the 259 women initially selected, 17 were lost to follow-up due to non-response, representing a rate of 6.56% (95% CI: 3.56–9.56%). This attrition level remains within acceptable limits for longitudinal studies, maintaining the representativeness of the final sample. Although the exclusion of 28 women due to pregnancy or the postpartum period during the first year after surgery may interfere with frequency estimates, these exclusions may have been interpreted positively by the patients, as infertility is a common concern in women with endometriosis. Indeed, in this context, it is important to emphasize that, although complex, minimally invasive nerve-sparing complete excision of endometriosis demonstrates a favorable safety profile [38]. Furthermore, pregnancy rates—whether spontaneous or assisted—have been consistently higher following primary surgery than after reoperation [39].

The absence of an objective, comprehensive assessment of both pelvic floor and sexual function represents another limitation of our study. Furthermore, superficial dyspareunia occurs concurrently with deep dyspareunia in up to 40% of patients, and individuals may find it difficult to differentiate between these symptoms independently [40]. Indeed, an isolated assessment of deep dyspareunia fails to capture overall sexual health; notably, women with endometriosis often exhibit a stronger correlation with sexual distress than with physical dysfunction alone [41]. Therefore, a concurrent evaluation of pelvic floor dysfunction and sexual distress using validated questionnaires should be incorporated into future research.

Given the retrospective, observational design of this study, accessing certain crucial data points was challenging, resulting in notable missing information. This included records on the regular use of analgesics, specific adjuvant hormonal treatments, and detailed descriptions of comorbidities such as psychiatric conditions, painful bladder syndrome, and irritable bowel syndrome. Additionally, chronic occupational stress warrants consideration when managing patients with DE. Observational data [42] have demonstrated a significant correlation between burnout syndrome—specifically, a diminished sense of personal accomplishment—and a higher prevalence of dyspareunia, highlighting an association between occupational exhaustion and painful intercourse.

Limitations regarding external validity must also be highlighted. While this single-center study benefits from high internal consistency due to a stable, highly experienced surgical team and a well-defined patient cohort, these factors may limit the generalizability of our findings to different healthcare settings. Specifically, our results may not fully translate to public hospitals, populations from diverse socioeconomic backgrounds, or less specialized centers.

While the majority of patients experience an improvement in dysmenorrhea and deep dyspareunia following surgical therapy for endometriosis [14,15,20,43], relying solely on measures of central tendency (such as means and medians) to evaluate treatment response presents a methodological limitation. These aggregated metrics can obscure unfavorable individual outcomes, which, despite affecting a minority of the study population, warrant dedicated investigation. Researchers should understand the variations in treatment response, thereby driving continuous therapeutic refinement and optimizing outcomes for all patients.

Regarding the potential influence of postoperative hormone use, of the 34 cases presenting with unfavorable outcomes, only 11 were using hormone therapy at the 1-year follow-up. Given that multiple determinants influence the persistence or onset of post-surgical deep dyspareunia, hormone use represents a critical candidate variable to be tested in future multivariate models.

We suggest that the analysis of surgical outcomes related to endometriosis should include long-term follow-up, as some symptoms might be transient. Future studies should include analysis of sexual function and other pathologies that may affect the development of endometriosis. (e.g., pelvic floor tension, painful bladder syndrome, and irritable bowel syndrome).

This study was limited to clinical and surgical parameters, and no immunological factors were evaluated. While incorporating biomolecular or inflammatory markers was beyond the scope of this clinical cohort (there is no data available), evaluating the interplay between immune responses and long-term deep dyspareunia outcomes could be an important target for future investigations. Blending clinical follow-up with the evaluation of these underlying immunological mechanisms, as discussed in recent literature [44], will be essential to fully elucidate pain persistence.

## 5. Conclusions

Even after analyzing individual cases through a semi-qualitative approach to generate hypotheses, reliable predictors of unfavorable outcomes regarding deep dyspareunia could not be identified. Therefore, this study highlights the marked phenotypic and clinical heterogeneity in individual patient trajectories, as well as the inherent unpredictability of adverse responses—such as the development of *De Novo* deep dyspareunia following surgery. These findings reinforce the necessity of a personalized medicine framework to guide researchers, clinicians, and patients through the uncertainties of preoperative counseling, supporting the development of individualized care pathways tailored to unique risk profiles. Ultimately, while minimally invasive nerve-sparing complete excision remains an effective and safe option for treating endometriosis-related deep dyspareunia, our findings corroborate the idea that even preoperatively asymptomatic patients must receive specific counseling regarding the unexpected risk of developing deep dyspareunia postoperatively.

## Figures and Tables

**Table 1:** Demographic characteristics prior to surgery (N = 195). The sample was dichotomized according to the severity of the deep dyspareunia reported prior to surgery: NONE or MILD (NRS ≤ 3) or MODERATE or SEVERE (NRS > 3).

		NONE MILD	N = 96		MODERATE SEVERE	N = 99		
		**N**	**%**		**N**	**%**		***p* Value**
Ethnicity	Asiatic	0	0	[4]	1	1.0	[1]	0.030
(self reported)	African descent	4	4.3		14	14.3		
	European descent	75	81.5		61	62.2		
	Indigenous	0	0		1	1.0		
	Mixed	13	14.5		21	21.4		
Partner	Never	18	18.8		16	16.2		0.651
(stable relationship)	Not currently	11	11.4		7	7.1		
	Yes	67	69.8		76	76.8		
Schooling	High school	1	1.1	[1]	0	0		0.658
(completed degree)	High school (completed)	6	6.3		12	12.1		
	College	10	10.5		12	12.1		
	College (completed)	31	32.6		30	30.3		
	Post-grad	4	4.2		3	3.0		
	Post-grad (completed)	43	45.3		42	42.4		
Income (U$/year)	<10,000	1	1.1	[2]	2	2.1	[2]	0.886
	10 to 20,000	8	8.5		12	12.4		
	20 to 50,000	35	37.2		35	36.1		
	50 to 100,000	32	34.0		30	30.9		
	>100,000	18	19.1		18	18.6		
Smoking	Never	88	96.7	[5]	88	90.7	[2]	0.083#
	≥5 cigarettes/day	1	1.0	[5]	4	4.1		0.069#
		10th	Median	90th	10th	Median	90th	
Alcohol consumption		0	0	2 [7]	0	0	2 [18]	0.929
Physical activity		0	2	4	0	2	4	0.742
Height (cm)		1.56	1.63	1.72	1.55	1.63	1.72	0.252
BMI (kg·m^−2^)		21	25	30	20	24	31	0.150
Age (years)		30.6	39.9	49.6	29.2	35.5	46.4	0.002

NRS: self-reported 11-point (0–10) numeric rating scale. Nonparametric independent-samples Mann-Whitney U test (2-sided) was used to compare groups according to ordinal variables. Pearson Chi-square test (2-sided) was used to compare groups according to categorical variables [#Fisher’s Exact Test]. Number of cases with missing data between brackets (question not answered). Income landmarks represent the total annual household income and were based on approximated values in December 2022. Alcohol consumption: days a week. Physical activity: days a week. BMI at 1-year follow-up. Age at 1-year follow-up.

**Table 2:** Systematic description of the surgical procedures concerning the main sites of endometriosis in the 34 women who presented undesirable response for deep dyspareunia to minimally invasive nerve-sparing complete excision of endometriosis at 1-year follow-up.

Cluster	Tube L/R	Ovary L/R	Uterus	RL L/R	Blad	Uret L/R	Param L/R	PF	Nerve L/R	USL L/R	Vag	RCerv	Sept	Bowel	Barr	Dur (min)
NONE (*De Novo*)																
1	P/P	EF/F		L/R	P	L/	Xd/Xd			L/R	X		X	Sh/Ap	F	
2	B/B	EPF/F	P				X/			L/R		X			C	90
3	B/B				P		X/X		/Ob + LS	L/R	X	X		Sh	F	165
4	P/B		A				/Xd			L/R					C	168
5	B/B	PF/								L/					C	37
6	P/P	F/PF				/R	/X			L/R	X		X	Dc	F	150
7	BS/P	F/	A	L/R	Sut		X/		Hn/	L/		X		Sh	F	180
8	S/S		Ht							L/R					C	65
9	P/P	EPF/EPF	M		Sut		/X			L/R	X	X		Sh	F	180
10	S/S	F/F	Ht							L/						70
11	S/S		Ht				/Xd		/Hn	/R			X			90
12	P/P	PF/P								L/R		X	X		F	65
13	P/P	EF/F			P	L/	X/		Hn/	L/R		X		Dc/Ap		
MODERATE (Nonresponder)																
14	P/P	F/					Xd/Xd	/M	/Hp	L/R		X	X		C	99
15	P/S	PF/PF	A/M/P							L/R		X			C	180
16	B/B	F/F	M												C	60
17	P/F	EPF/	P/Mh	L/R	P	L/	X/			L/R	X	X			F/C	180
18	P/P	/F					X/X			L/R	X	X			F	90
19	B/B	EPF/F	A/Syn	L/	P			M/		L/R	X	X		Dc	F	180
20	P/B	F/	A	L/	P		/Xd			L/R	X	X			F	
21	B/P	F/F	M							L/R		X	X			
22	P/B	EPF/EPF	A		Sut	L/R	Xd/X		Hn/Hn	L/R	X	X		Sh/Dc	F	330
MODERATE (Paradoxal)																
23	HS/HS	F/F	Ht	L/R			/X			L/					C	160
SEVERE (Nonresponder)																
24	P/B	F/F								L/R	X	X	X	Seg	F	
25	S/	P/F	Ht							/R					F	
26	S/S	F/F	Ht				/X					X			F	105
27	P/P	F/	M				/Xd								F	
28	P/P	EPF/F	M			L/	X/X		Hn/Hn	L/R		X		Dc	F	95
29	P/B	P/P					/Xd	Ss/	/Hn	L/R		X			F	58
30	S/S	P/	Ht		P					L/R		X			F	162
31	P/P						/Xd			L/R	X	X		Ap	C	120
32	HS/HS	F/	A	L/R	P		X/X				X	X		Sh	C	164
33	P/B	EPF/F			P	L/	/Xd			L/R	X	X			C	180
34	S/HS	EPF/F	Ht		P		X/Xd			L/R		X		DDc	F	200

Tube: P pervious or B blocked at chromopertubation; S salpingectomy; H hydrosalpingix; N.A. not applicable (previous salpingectomy). Ovary: T oophorectomy; P oophoroplasty; E endometrioma; F presence of endometriosis in the peritoneum of the ovarian fossa. Uterus: Ht total hysterectomy; M myomectomy; A adenomyomectomy; P hysteroscopic polipectomy; Mh hysteroscopic myomectomy; Syn Hysteroscopic synechiolysis; N.A. not applicable (previous hysterectomy). RL (Round ligament). Blad (Bladder): Sut partial cystectomy and intracorporeal suturing; P superficial nodule infiltrating bladder peritoneum “shaving”. Uret means ureterolysis (systematic procedure aimed at exposing the ureter in order to free it from external pressure or adhesions). Param (Parametrium): Xd means deeper resection, below the ureter (paracolpium). PF (pelvic floor): Ss sacrospinous ligament; M pelvic floor muscle. Nerve (excision of endometriosis nodule infiltrating nerve): Hn hypogastric nerve; Hp inferior hypogastric plexus; Obt: obturator nerve; LS: lumbosacral trunk. USL (Uterosacral ligament). Vagina: endometriotic nodule excision of vagina. Rcerv (Retrocervical). Sept (Rectovaginal septum). Bowel: Ap appendicectomy; Seg segmental Shav shaving; Disc discoid, DDisc double discoid resection. Barr (Barrier agents for adhesion prevention): F fibrin sealant (human); C carboxymethylcellulose. L/R: Left/Right. Dur: Surgery duration. *De Novo*: Unexpected postoperative onset of deep dyspareunia. Nonresponder: deep dyspareunia relief of < 3 points. Paradoxal: relevant worsening of deep dyspareunia at the 1-year follow-up (≥3 points).

**Table 3:** Possible covariates in the assessment of deep dyspareunia. Data from 34 women who presented undesirable response of deep dyspareunia to minimally invasive nerve-sparing complete excision of endometriosis at 6-month and 1-year follow-up.

Cluster	Dysp (NRS)	Dysm (NRS)	PPain (NRS)	Length of PPain	Previous Pelvic Surgery	Comorbidities	Indication of Surgery	Previous Hormonal Therapy	Hormonal Therapy at 1 Year Follow-Up	Goserr
NONE (*De Novo*)										
1	0-9-6	10-NA	4-0	1			Pain	None	cCOC	2
2	0-?-8	10-NA	0-2			Depression ^#^	Pain	None	LNG-IUS	1
3	0-2-3	10-0	2-0	9			Pain + Inf	None	None	
4	0-0-5	0-NA	0-0	0	Endo	Depression ^#^	Pain	ENG	ENG	
5	0-0-3	8-NA	8-2	1			Pain	OP		2
6	0-0-3	8-NA	0-0	0			Pain	OP		2
7	0-0-3	NA-NA	8-0	18	Endo	PID	Pain + Inf	OP		1
8	0-2-3	NA-NA(Ht)	4-0	24			Image	cCOC		
9	0-0-5	9-0	0-0	0			Pain	None	None	
10	0-0-3	8-NA(Ht)	0-3	0			Pain	None	None	
11	0-0-5	NA-NA(Ht)	8-0	10			Pain	OP	None	
12	0-4-3	8-3	5-4	12			Inf	None	None	
13	0-2-3	10-NA	0-8	0			Pain-Inf	OP	cCOC	1
MODERATE (Nonresponder)										
14	4-4-2	NA-NA	0-0	0	Urologic	Depression ^#^ and Migraine		LNG-IUS	LNG-IUS	
15	6-4-7	10-4	4-7	6	Colorectal		Pain + Inf	None	None	
16	6-0-4	7-NA	4-5	36		Vulvodynia	Pain	cCOC		2
17	5-?-6	10-1	5-0	36		Intersticial Cystitis	Pain + Inf	None	None	
18	4-4-2	1-6	0-0	0		Hypothyroidism	Inf	None	None	
19	5-0-3	NA-NA	8-3	24			Pain	VR	OP	
20	6-5-6	8-NA	6-4	200		Endometritis	Pain	None	None	
21	5-5-4	NA-NA	0-0	0			Image	cCOC	cCOC	
22	4-5-6	7-1	10-0	3		Oophoroplasty	Pain-Inf	None	None	
MODERATE (Paradoxal)										
23	5-8-8	9-NA(Ht)	8-9	3		Depression ^#^ and Diabetes	Pain	None		
SEVERE (Nonresponder)										
24	10-?-9	10-NA	0-0	0		Vaginismus	Pain	OP	cCOC	
25	10-2-10	8-NA	9-0	24	Right SO		Pain	LNG-IUS	LNG-IUS	
26	9-9-9	NA-NA(Ht)	9-8	36			Pain	OP	None	
27	10-?-10	10-10	10-10	99		PLLLP	Pain	None	None	1
28	10-?-9	10-4	7-0	24			Pain-Inf	None	None	
29	7-0-6	NA-NA	9-1	24			Pain	cCOC		
30	8-4-8	0-NA(Ht)	0-0	0		Hypertension	AUB	None	None	
31	9-7-9	NA-4	9-0	7			Pain	cCOC	None	
32	7-8-5	NA-NA	4-2	72			Pain	OP	cCOC	
33	8-6-8	8-NA	6-5	24			Pain	LNG-IUS	COCcyc	
34	9-8-10	NA-NA(Ht)	4-8	48			Pain	None	None	2

NRS: 11-point self-reported Numerical Rating Scale for pain assessment. Dysp: Deep Dyspareunia scores (prior to surgery—at 6 months—at 1 year). Dysm: Dysmenorrhea scores (prior to surgery—at 1 year). PPain: non-cyclical pelvic pain scores (prior to surgery—at 1 year); Length of PPain in months. Ht: hysterectomy. Right SO: laparotomic right salpingo-oophorectomy due to ovarian torsion. Endo: previous endometriosis surgery. PID: Pelvic inflammatory disease. PLLLP: Postoperative lower left limb paresthesia. AUB: abnormal uterine bleeding. Inf: infertility. Image: radiologic findings like endometriomas > 5 cm, ureteral or bowel stenosis. LNG-IUS levonorgestrel-releasing intrauterine system. ENG: etonogestrel subdermal implant. OP: oral progesterone. cCOC: continuous combined oral contraceptive. COCcyc: cyclical combined oral contraceptive. VR: vaginal ring. Goserr: Goserrelin implant (number of doses). #Continuous psychotropic medications: Case 2: Major depression (sertraline, topiramate, and medical cannabis); Case 4: Major depression (zolpidem, diazepam, lisdexamfetamine, agomelatine, gabapentin); Case 14: Major depression (specific pharmacological agents were not informed); Case 23: Major depression (specific pharmacological agents were not informed). *De Novo*: Unexpected postoperative onset of deep dyspareunia. Nonresponder: deep dyspareunia relief of < 3 points. Paradoxal: relevant worsening of deep dyspareunia at the 1-year follow-up (≥3 points).

## Data Availability

The original contributions presented in this study are included in the article/Supplementary Material. Further inquiries can be directed to the authors.

## Supplementary Materials

The following supporting information can be downloaded at: https://www.mdpi.com/article/10.3390/jpm16060307/s1.

## References

[1] Zondervan K.T., Becker C.M., Missmer S.A. (2020). Endometriosis. N. Engl. J. Med..

[2] Facchin F., Barbara G., Saita E., Mosconi P., Roberto A., Fedele L., Vercellini P. (2015). Impact of endometriosis on quality of life and mental health: Pelvic pain makes the difference. J. Psychosom. Obstet. Gynaecol..

[3] Daniilidis A., Angioni S., Di Michele S., Dinas K., Gkrozou F., D’Alterio M.N. (2022). Deep Endometriosis and Infertility: What Is the Impact of Surgery?. J. Clin. Med..

[4] Roomaney R., Mitchell H. (2022). Psychosocial correlates of symptoms of depression among patients with endometriosis in the United Kingdom. Women Health.

[5] Wang P.H., Yang S.T., Chang W.H., Liu C.H., Lee F.K., Lee W.L. (2022). Endometriosis: Part I. Basic concept. Taiwan. J. Obstet. Gynecol..

[6] De Graaff A.A., D’Hooghe T.M., Dunselman G.A., Dirksen C.D., Hummelshoj L., Simoens S., Bokor A., Brandes I., Brodszky V., WERF EndoCost Consortium (2013). The significant effect of endometriosis on physical, mental and social wellbeing: Results from an international cross-sectional survey. Hum. Reprod..

[7] Fritzer N., Haas D., Oppelt P., Renner S., Hornung D., Wölfler M., Ulrich U., Fischerlehner G., Sillem M., Hudelist G. (2013). More than just bad sex: Sexual dysfunction and distress in patients with endometriosis. Eur. J. Obstet. Gynecol. Reprod. Biol..

[8] Becker C.M., Bokor A., Heikinheimo O., Horne A., Jansen F., Kiesel L., King K., Kvaskoff M., Nap A., Petersen K. (2022). ESHRE guideline: Endometriosis. Hum. Reprod. Open.

[9] Abrao M.S., Petraglia F., Falcone T., Keckstein J., Osuga Y., Chapron C. (2015). Deep endometriosis infiltrating the recto-sigmoid: Critical factors to consider before management. Hum. Reprod. Update.

[10] Collinet P., Fritel X., Revel-Delhom C., Ballester M., Bolze P.A., Borghese B., Bornsztein N., Boujenah J., Brillac T., Chabbert-Buffet N. (2018). Management of endometriosis CNGOF/HAS clinical practice guidelines short version. J. Gynecol. Obstet. Hum. Reprod..

[11] Oliveira M.A., Pereira T.R., Gilbert A., Tulandi T., de Oliveira H.C., De Wilde R.L. (2016). Bowel complications in endometriosis surgery. Best. Pract. Res. Clin. Obstet. Gynaecol..

[12] Gasparoni M.P., de Freitas Fonseca M., Favorito L.A., da Silva Filho F.S., Diniz A.L.L., Schuh M.F., Gomes F.H., de Resende J.A.D. (2024). Unilateral nerve preservation during parametrectomy is not sufficient to prevent persistent urinary retention after cytoreductive endometriosis surgery. Arch. Gynecol. Obstet..

[13] Gomes F.H., Fonseca M.F., Favorito L.A., Gasparoni M.P., da Silva Filho F.S., Diniz A.L.L., de Resende J.A.D. (2024). Changes in lower urinary tract function after minimally invasive nerve-sparing for complete excision of endometriosis: An observational study. Neurourol. Urodyn..

[14] Parra R.S., Feitosa M.R., Camargo H.P., Valério F.P., Zanardi J.V.C., Rocha J.J.R.D., Féres O. (2021). The impact of laparoscopic surgery on the symptoms and wellbeing of patients with deep infiltrating endometriosis and bowel involvement. J. Psychosom. Obstet. Gynaecol..

[15] Zhang N., Sun S., Zheng Y., Yi X., Qiu J., Zhang X., Zhang Y., Hua K. (2022). Reproductive and postsurgical outcomes of infertile women with deep infiltrating endometriosis. BMC Womens Health.

[16] Fritzer N., Tammaa A., Haas D., Oppelt P., Renner S., Hornung D., Wölfler M., Ulrich U., Hudelist G. (2016). When sex is not on fire: A prospective multicentre study evaluating the short-term effects of radical resection of endometriosis on quality of sex life and dyspareunia. Eur. J. Obstet. Gynecol. Reprod. Biol..

[17] Kent A., Shakir F., Rockall T., Haines P., Pearson C., Rae-Mitchell W., Jan H. (2016). Laparoscopic Surgery for Severe Rectovaginal Endometriosis Compromising the Bowel: A Prospective Cohort Study. J. Minim. Invasive Gynecol..

[18] Vercellini P., Frattaruolo M.P., Rosati R., Dridi D., Roberto A., Mosconi P., De Giorgi O., Cribiù F.M., Somigliana E. (2018). Medical treatment or surgery for colorectal endometriosis? Results of a shared decision-making approach. Hum. Reprod..

[19] Bafort C., Dancet E., Mellaerts J., Meuleman C., Tomassetti C. (2024). Correlation Between Surgical Phenotype and Pain Improvement After Endometriosis Surgery. J. Minim. Invasive Gynecol..

[20] Crispi C.P., Crispi C.P., de Oliveira B.R.S., de Nadai Filho N., Peixoto-Filho F.M., Fonseca M.F. (2021). Six-month follow-up of minimally invasive nerve-sparing complete excision of endometriosis: What about dyspareunia?. PLoS ONE.

[21] von Elm E., Altman D.G., Egger M., Pocock S.J., Gøtzsche P.C., Vandenbroucke J.P., STROBE Initiative (2008). The Strengthening the Reporting of Observational Studies in Epidemiology (STROBE) statement: Guidelines for reporting observational studies. J. Clin. Epidemiol..

[22] Agha R.A., Sohrabi C., Mathew G., Franchi T., Kerwan A., O’Neill N., PROCESS Group (2020). The PROCESS 2020 Guideline: Updating Consensus Preferred Reporting Of CasE Series in Surgery (PROCESS) Guidelines. Int. J. Surg..

[23] Mathew G., Sohrabi C., Franchi T., Nicola M., Kerwan A., Agha R., PROCESS Group (2023). Preferred Reporting of Case Series in Surgery (PROCESS) 2023 guidelines. Int. J. Surg..

[24] Dindo D., Demartines N., Clavien P.A. (2004). Classification of surgical complications: A new proposal with evaluation in a cohort of 6336 patients and results of a survey. Ann. Surg..

[25] Gerlinger C., Schumacher U., Faustmann T., Colligs A., Schmitz H., Seitz C. (2010). Defining a minimal clinically important difference for endometriosis-associated pelvic pain measured on a visual analog scale: Analyses of two placebo-controlled, randomized trials. Health Qual. Life Outcomes.

[26] Carli F. (2015). Physiologic considerations of Enhanced Recovery After Surgery (ERAS) programs: Implications of the stress response. Can. J. Anaesth..

[27] Kim T.K., Yoon J.R. (2010). Comparison of the neuroendocrine and inflammatory responses after laparoscopic and abdominal hysterectomy. Korean J. Anesthesiol..

[28] McNamara H.C., Frawley H.C., Donoghue J.F., Readman E., Healey M., Ellett L., Reddington C., Hicks L.J., Harlow K., Rogers P.A.W. (2021). Peripheral, Central, and Cross Sensitization in Endometriosis-Associated Pain and Comorbid Pain Syndromes. Front. Reprod. Health.

[29] Ianieri M.M., Raimondo D., Rosati A., Cocchi L., Trozzi R., Maletta M., Raffone A., Campolo F., Beneduce G., Mollo A. (2022). Impact of nerve-sparing posterolateral parametrial excision for deep infiltrating endometriosis on postoperative bowel, urinary, and sexual function. Int. J. Gynaecol. Obstet..

[30] Vercellini P., Somigliana E., Consonni D., Frattaruolo M.P., De Giorgi O., Fedele L. (2012). Surgical versus medical treatment for endometriosis-associated severe deep dyspareunia: I. Effect on pain during intercourse and patient satisfaction. Hum. Reprod..

[31] Velho R.V., Taube E., Sehouli J., Mechsner S. (2021). Neurogenic Inflammation in the Context of Endometriosis—What Do We Know?. Int. J. Mol. Sci..

[32] Bair M.J., Robinson R.L., Katon W., Kroenke K. (2003). Depression and pain comorbidity: A literature review. Arch. Intern. Med..

[33] Del Pino-Sedeño T., Cabrera-Maroto M., Abrante-Luis A., González-Hernández Y., Ortíz Herrera M.C. (2024). Effectiveness of psychological interventions in endometriosis: A systematic review with meta-analysis. Front Psychol..

[34] Fonseca M.F., Sessa F.V., Crispi C.P., Filho N.N., de Oliveira B.R.S., Garcia R.F., Crispi C.P. (2025). Non-menstrual pelvic symptoms and women’s quality of life: A cross-sectional observational study. PLoS ONE.

[35] Collinet P., Renso M., Briez N. (2025). Do we need a preventive stoma in surgery for colorectal endometriosis? A retrospective series of 97 patients treated at an expert centre. Facts Views Vis. Obgyn.

[36] Crispi C.P., Crispi C.P., Joaquim C.M.V., Reis P.S.D.S., de Nadai Filho N., de Oliveira B.R.S., Guerra C.G.S., Fonseca M.F. (2025). Follow-up of bowel endometriosis resections performed using the double circular stapler technique: A decade’s experience. PLoS ONE.

[37] Sinha R., Rupa B., Raina R., Bag M. (2025). Comparison of Laparoscopic and Robotic Intraoperative Adverse Events in Benign Gynecological Procedures and the Correlation of the Adverse Events with Postoperative Outcomes and Risk Analysis. Cureus.

[38] Oliveira B.R., Crispi C.P., Russomano F.B., Peixoto Filho F.M., de Nadai Filho N., Fonseca M.F. (2026). Safety and Reproductive Outcomes of Minimally Invasive Nerve-Sparing Surgery for Deep Endometriosis in Infertile Women: A One-Year Follow-Up Study. J. Pers. Med..

[39] Cao M., Koroneos Z., Tyson K., Mooney S., Holdsworth-Carson S. (2026). Fertility after endometriosis surgery: A systematic review and meta-analysis comparison of primary versus multiple surgical interventions. Eur. J. Obstet. Gynecol. Reprod. Biol..

[40] Yong P.J. (2017). Deep Dyspareunia in Endometriosis: A Proposed Framework Based on Pain Mechanisms and Genito-Pelvic Pain Penetration Disorder. Sex. Med. Rev..

[41] Rossi V., Galizia R., Tripodi F., Simonelli C., Porpora M.G., Nimbi F.M. (2022). Endometriosis and Sexual Functioning: How Much Do Cognitive and Psycho-Emotional Factors Matter?. Int. J. Environ. Res. Public Health.

[42] Pham T.H., Vo M.T., Nguyen P.N. (2025). Burnout Syndrome and Sexual Disorders Among Vietnamese Female Nurses and Midwives at Tu Du Hospital: A Frontline Hospital-Based Cross-Sectional Study. Women’s Health Rep..

[43] Hudelist G., Darici Kurt E., Szabó G., Miklos D., Hudelist T., Bokor A. (2025). Surgical outcomes of women undergoing radical resection of deep endometriosis of the sacral plexus: A prospective cohort study. Acta Obstet. Gynecol. Scand..

[44] Laudański P., Rogalska G., Warzecha D., Lipa M., Mańka G., Kiecka M., Spaczyński R., Piekarski P., Banaszewska B., Jakimiuk A. (2023). Autoantibody screening of plasma and peritoneal fluid of patients with endometriosis. Hum. Reprod..

